# How does invasion degree shape alpha and beta diversity of freshwater fish at a regional scale?

**DOI:** 10.1002/ece3.9493

**Published:** 2022-11-08

**Authors:** Anna Gavioli, Marco Milardi, Janne Soininen, Elisa Soana, Mattia Lanzoni, Giuseppe Castaldelli

**Affiliations:** ^1^ Department of Environmental and Prevention Sciences University of Ferrara Ferrara Italy; ^2^ Fisheries New Zealand ‐ Tini a Tangaroa, Ministry for Primary Industries ‐ Manatū Ahu Matua Wellington New Zealand; ^3^ Department of Geosciences and Geography University of Helsinki Helsinki Finland; ^4^ Present address: Southern Indian Ocean Fisheries Agreement (SIOFA) Saint‐Denis Cedex La Réunion

**Keywords:** beta diversity, biodiversity, human impact, invasive species, LCBD, non‐native species, species richness

## Abstract

Freshwater ecosystems appear more vulnerable to biodiversity loss due to several anthropogenic disturbances and freshwater fish are particularly vulnerable to these impacts. We aimed to (1) identify the contribution of land use, spatial variables, and invasion degree in determining freshwater fish alpha (i.e., species richness) and beta (i.e., local contributions to beta diversity, LCBD) diversity, evaluating also the relationship between invasion degree and nestedness (β_nes_) and turnover (β_sim_) components of beta diversity. (2) Investigate the relationship between alpha diversity and LCBD, under the hypothesis that alpha diversity and LCBD correlate negatively and (3) investigate the relationship between species contributions to beta diversity (SCBD) and species occurrence, hypothesizing that non‐native species show a lower contribution to beta diversity. The linear mixed models and the partition of *R* ^2^ retained the invasion degree as the most important variables explaining alpha and beta diversity, having a positive relationship with both diversity components. Furthermore, land use related to human impacts had a positive influence on alpha diversity, whereas it showed a negative effect on LCBD. Regression model further showed that invasion degree related positively with β_sim_, but negatively with β_nes_, suggesting that non‐native species were involved in the replacement of native species in the fish community. Alpha diversity and LCBD showed a weak positive correlation, meaning that sites with low species richness have higher LCBD. SCBD scaled positively with species occurrence highlighting that rarer species contribute less to SCBD. Finally, native and exotic species contributed similarly to beta diversity. These results suggest that invasion degree plays a central role in shaping alpha and beta diversity in stream fish, more than land use features reflecting habitat alteration or other geospatial variables. Furthermore, it is important to evaluate separately the native and the non‐native components of biotic communities to identify linkages between invasion dynamics and biodiversity loss.

## Graphical abstract

Non‐native species presence plays a central role in shaping alpha and beta diversity patterns. Land uses related to human impacts showed positive influence of alpha fish diversity, whereas beta diversity was negatively influenced.

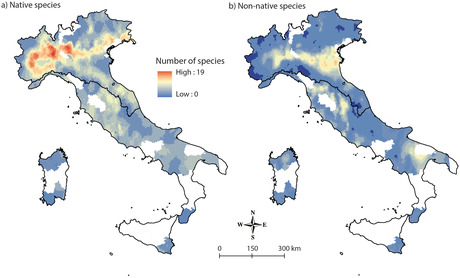

## 1 INTRODUCTION

Biodiversity is not equally distributed on Earth but shows geographical patterns (Gaston, 2000; Hillebrand, 2004), which are being altered by global environmental changes. Due to its great variability, understanding the distribution of biodiversity has important implications in conservation and management plans, in studying species' niches, in the assessment of anthropogenic impacts (e.g. climate change and land use), and in the investigation of biological invasion dynamics (see e.g. Guisan & Thuiller, 2005).

Although the study of biodiversity changes across communities is not an easy task due to its scale‐dependence (Chase et al., 2018), the most common way to investigate biodiversity patterns is the study of variations in taxonomical species diversity (Colwell & Coddington, 1994). Three different levels of taxonomical diversity can be distinguished: alpha (i.e., local diversity), beta (i.e., variation of community composition among sites), and gamma diversity (i.e., regional diversity; Whittaker, 1960, 1972). Several measures were proposed to investigate each level of diversity: for example, the Shannon–Wiener index and species richness (see e.g. Magurran, 2004) for alpha diversity, and the turnover and nestedness components of beta diversity (Baselga, 2010). Alternatively, beta diversity can be characterized as the variance of community data, which can be partitioned into local contributions to beta diversity (LCBD) and species contributions to beta diversity (SCBD) (Legendre & De Cáceres, 2013). As LCBD represents the uniqueness of sites based on community variation, different environmental variables such as altitude and catchment size can determine LCBD values (Tonkin et al., 2016). SCBD is associated with species characteristics, such as abundance and occurrence (Heino & Grönroos, 2017). Finally, the total effective number of species in the data set can be used to assess gamma diversity (see e.g. Tuomisto, 2010).

Different spatial and environmental factors combine to determine global diversity patterns. Worldwide, species diversity varies across latitudinal gradients with more species close to the equator than the poles and across altitude with a general decrease of species from low to high altitudes (Gaston, 2000; Gaston et al., 2008). For example, most fish communities experience diversity loss with increasing altitude due to the increase of environmental harshness and decrease in the available habitat area (Jaramillo‐Villa et al., 2010). However, different anthropogenic pressures can also affect diversity patterns, usually leading to biodiversity loss either in terms of decreasing richness or increasing community similarity (Butchart et al., 2010; Ceballos et al., 2015; Dirzo et al., 2014; Gavioli et al., 2019).

Freshwater ecosystems, which host a large number of endemic and rare species (Balian et al., 2008; Collen et al., 2014; Gleick, 1998), appear vulnerable to many anthropogenic pressures like species introduction, flow regulation, land use change, pollution, overexploitation, and climate change (Carpenter et al., 2011; Dudgeon, 2019; Olden & Rooney, 2006; Rahel & Olden, 2008; Vörösmarty et al., 2010). In freshwaters, non‐native species are responsible for the decline of native fish species population (Costa et al., 2021; Crivelli, 1995; Hermoso et al., 2011), and fish species are one of the most introduced taxa worldwide (Gozlan et al., 2010). Despite the large number of introduced fish species, only a subset of these species can establish viable populations in the new environment (Jeschke & Strayer, 2005) and become invasive (Colautti & MacIsaac, 2004; Leprieur et al., 2008). The main mechanisms through which non‐native species can affect native ones include predation, competition, decreasing genetic heterogeneity, and habitat alteration (e.g. Ribeiro & Leunda, 2012; Simberloff et al., 2013). Non‐native fish introductions can drive biotic homogenization of communities, a process whereby communities become more similar over time due to the combined effects of native species loss and non‐native species introductions (Olden et al., 2010; Rahel, 2000). As a consequence, locally representative fish species (e.g., endemic species, habitat specialists) are replaced by cosmopolitan species (Rahel, 2007, 2010).

Recently, considerable effort has been put into assessing diversity changes (e.g. in alpha and beta diversity) in freshwater environments (e.g. Edge et al., 2017; Giovâni da Silva et al., 2018), however, some knowledge gaps remain about the different pressures on diversity, since their effects overlap in space and time and cannot be easily disentangled. Despite the importance of understanding these mechanisms for example for conservation and management purpose, their study requires data sets that have large spatial extent encompassing different communitis. Here, we focused on the Mediterranean region, as it is one of the biodiversity hotspots identified by Myers et al. (2000), where native biodiversity, including several endemic species, is at risk from biological invasions (Hermoso et al., 2011; Marr et al., 2010). We focused on freshwater fish as model taxa due to their susceptibility to anthropogenic impacts (Closs et al., 2015; Dudgeon, 2019).

The contributions of land use features (as a proxy for habitat exploitation), geospatial variables, and invasion degree (i.e., the abundance‐based share of introduced species of the total community at each sampling site) to the freshwater fish diversity patterns were investigated using a fine‐scale resolution fish data extended throughout the Italian peninsula.

Non‐native fish species have negative effect on fish diversity (e.g., Clavero & García‐Berthou, 2005); thus, we hypothesized that (H1) invasion degree is the strongest driver negatively influencing alpha diversity and LCBD in the overall fish community. We also investigated how invasion degree affects different beta diversity components (turnover and nestedness).

Typically, alpha diversity and LCBD have a negative relationship, indicating that sites with unique species composition harbor low species richness (e.g. Legendre & De Cáceres, 2013). However, such relationship varies depending on the region and the spatial extent covered (Dansereau et al., 2022). We investigated the relationship between alpha diversity and LCBD hypothesizing that (H2), in the fish communities in Italy, sites with unique species composition (higher value of LCBD) also show lower alpha diversity due to the presence of rare species, which contribute to higher value of LCBD (Giovâni da Silva et al., 2018). Finally, in order to evaluate the different contributions of native and non‐native species to beta diversity, we investigated the relationship between SCBD and species occurrence. We hypothesized (H3), that non‐native species show a lower contribution to beta diversity (lower values of SCBD) compared to native species, because most non‐native species are cosmopolitan having wide occurrence also regionally (Rahel, 2000), thus contributing only little to the variation of the community between regions.

## 2 METHODS

### 2.1 Data collection

Freshwater fish community data in Italian watercourses were obtained from Milardi et al. (2020) with a total of 3734 sites, covering most of the Italian peninsula and nearby islands, spanning altitudes from −4 m to 2556 m above sea level, collected through official monitoring programs. Fish sampling was mainly performed in the warm season by electrofishing, combined with nets in sites of higher water depth and conductivity as indicated in national monitoring guidelines (APAT, 2007). More details on fish sampling methodology can be found in Lanzoni et al. (2018) and Milardi et al. (2018).

Sampling time spanned the years 1999–2014; however, fish communities are typically more or less stable over such timescales (Korhonen et al., 2010), and the data were collected within a relatively short timeframe (typically within 7 years) within each district (Gavioli et al., 2019); thus, time presumably did not affect notably our result. Furthermore, non‐native species introductions in Italy occurred long before the sampling period (e.g., common carp *Cyprinus carpio* was introduced in the 17th century, and North American species such as brown bullhead *Ameiurus melas* were introduced in the early 19th century).

Fish species were classified according to Kottelat and Freyhof (2007), taking into account recent taxonomic determinations and common names as listed in FishBase (Froese & Pauly, 2019).

Species were categorized as native or introduced species according to their biogeographic origin, as established through the current scientific literature (e.g. IUCN, 2021).

**FIGURE 1:**
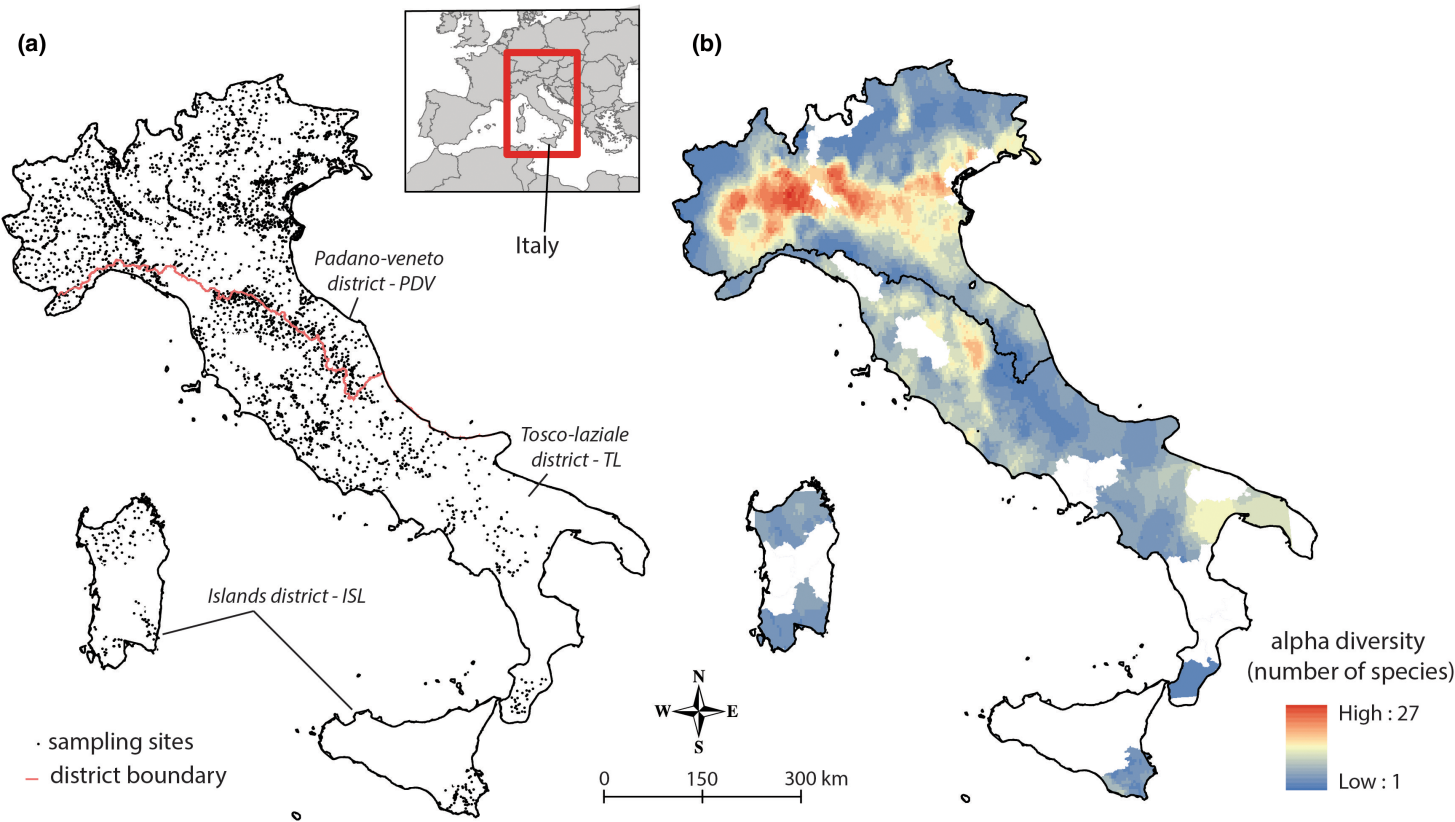
Sampling sites within biogeographical districts (a) and alpha diversity (i.e., number of species) (b) of fish communities in Italian inland waters. White areas in (b) represent zones for which no fish data was collected.

Based on scientific literature (Bianco, 1987, 1998), three biogeographical districts separated from each other by geographical barriers (i.e., mountain chains or sea stretches) were distinguished to account for non‐native species introduction (Figure 1): the Padano‐Veneto district in northern Italy (PDV, 2418 sites, ~126.000 km^2^), which includes the largest river basin in Italy (i.e. the Po River basin), the Tosco‐Laziale district in central and southern Italy (TL, 1146 sites, ~124.000 km^2^), and the Islands district (ISL, 170 sites, equally divided between the islands of Sardinia and Sicily, ~49.000 km^2^).

According to (Milardi et al., 2020), species was defined as introduced when introduction was human mediated. These include species originated from outside of the Italian geographical barriers (i.e. exotic species) and native species introduced from one district to new areas (i.e. translocated species). Hybrid specimens or uncertain species were excluded from this study.

### 2.2 Diversity measures

Alpha diversity was investigated as species richness, and it was measured as the number of all fish species present at each sampling site (Whittaker, 1972). Beta diversity was assessed as the total variance of the fish community matrix following Legendre and De Cáceres (2013). This method partitions the total beta diversity (BD_total_) into Local Contributions to Beta Diversity (LCBD) (i.e., site contributions) and into Species Contributions to Beta Diversity (SCBD). The LCBD represents the uniqueness of fish community composition across sites: sites with higher values of LCBD indicate an unusual species composition compared with the average community. The SCBD shows the degree of variation of a species across sites, and it can be considered as a measure of the relative importance of each species in affecting beta diversity (Heino & Grönroos, 2017; Legendre & De Cáceres, 2013). To calculate LCBD and SCBD abundance‐based, the site by species abundance matrix was Hellinger transformed (Legendre & De Cáceres, 2013).

The different components of beta diversity (total beta diversity‐βsor, species turnover‐βsim, and nestedness‐βnes) were also investigated using Sorensen dissimilarity index (Baselga, 2010). The turnover component identifies the degree of species replacement between sites, whereas the nestedness component identifies the variation in species richness. Alpha diversity, LCBD, and SCBD measures were calculated using “vegan” (Oksanen et al., 2017) and “adespatial” (Dray et al., 2018) R packages, respectively. The nestedness and turnover components of beta diversity were calculated using “betapart” R package (Baselga et al., 2017).

### 2.3 Invasion degree, geospatial, and land features

For each sampling site, invasion degree was calculated as a of introduced species in fish communities, based on the abundance of native and non‐native fish data (see Milardi et al., 2020 for more details). Invasion degree expresses a corrected ratio of native/non‐native species, where the correction factors account for each species numerical abundance and species‐specific body size. As such, invasion degree is independent of diversity measures. A high invasion degree equals to a high share of introduced species (i.e. exotic and translocated species) and a low share of native species in terms of abundance within the fish community.

Geospatial variables (i.e. latitude, longitude, and altitude) and land use features for each sampling site's watershed were calculated through ArcGIS 10.1 software, using the CORINE database (2012, https://www.eea.europa.eu/data‐and‐maps/data/copernicus‐land‐monitoring‐service‐corine). In the lowland areas, where the low slopes and the human flow modification do not permit an easy definition of watershed areas, the land cover of the whole river basin or of the administrative province was used. CORINE land cover classes were merged into 13 categories: artificial surface, non‐irrigated arable land, irrigated arable land, rice field, permanent crop, pasture, heterogeneous agricultural area, forest, natural vegetated area, natural cover without vegetation, sand area, freshwater, and marine water. Land cover was expressed as the percent cover of each of these categories in the watershed of each site (Table 1).

**TABLE 1:** Abbreviations, units, statistics, and group of each explanatory variable.

Explanatory variables	Unit	Average	St.deviation	Group
Longitude	Dec. degrees	11.242	2.102	Geospatial variables
Latitude	Dec. degrees	44.050	2.045	Geospatial variables
Altitude	m a.s.l	301.815	318.937	Geospatial variables
Artificial surface	%	7.396	17.712	Land use
Nonirrigated arable land	%	23.101	32.830	Land use
Permanently irrigated land	%	0.004	0.186	Land use
Rice fields	%	1.796	11.369	Land use
Permanent crops	%	3.182	10.984	Land use
Pastures	%	1.524	5.904	Land use
Heterogeneous agricultural areas	%	14.816	21.035	Land use
Forest	%	33.514	32.481	Land use
Natural vegetated area	%	9.531	16.578	Land use
Sand areas	%	0.666	5.061	Land use
Natural cover without vegetation	%	2.147	8.228	Land use
Freshwaters	%	1.139	7.482	Land use
Marine waters	%	1.184	7.952	Land use
Abundance‐based share of introduced species	%	22.101	29.618	Invasion degree

### 2.4 Data analysis

The influence of invasion degree, geospatial, and land features (i.e. explanatory variables) on alpha diversity and LCBD (i.e. response variables) was evaluated through linear mixed models. Alpha diversity was log‐transformed and the explanatory variables were standardized (Philson et al., 2021).

Originally, we performed the linear mixed models including the river basins as random effects to account for spatial dependence (results are not shown here). However, due to the higher level of random effect (*n* = 129) and the nestedness of river basins inside the biogeographical districts, we decided to include in linear mixed models the biogeographical districts (District) as a random effect to account for spatial dependence. However, results were not divergent between the inclusion of river basins and District as random effect. Collinearity of explanatory variables was assessed through the variance inflation factor (VIF). To identify a set of explanatory variables without collinearity, one variable is removed at a time, the VIF values were recalculated, and the procedure was repeated until all VIF values were smaller than 5 (Zuur et al., 2009). As result, longitude, non‐irrigated arable land, natural vegetated area, and sand area variables were excluded from the models to avoid collinearity problems (VIF > 5).

The Akaike Information Criterion (AIC; Akaike, 1974) was used to select the best model among a set of possible candidate models. The selection of the best model was based on Akaike weights (models with large Akaike weights have strong support) and lowest AIC values (Snipes & Taylor, 2014).

To estimate the variance explained by each of the fixed and random effects of the best models selected, the marginal and conditional *R* ^2^ values were calculated for each linear mixed model (Stoffel et al., 2021). The marginal *R* ^2^ gives an estimate of the variance explained by each fixed effect relative to the total variance in the response, whereas the conditional *R* ^2^ gives an estimate of the variance explained by fixed effects and random effects together, which better reflects the heterogeneity of the variables. The 95% confidence intervals were estimated for the marginal and conditional *R* ^2^ using 1000 parametric bootstrap iterations (Stoffel et al., 2021).

Linear regression was used to investigate the relationship between invasion degree and the turnover (β_sim_) and the nestedness (β_nes_) components of beta diversity. As β_sim_ β_nes_ components are distance matrices, the invasion degree was converted into a Euclidean distance matrix to perform the model regression. As values of LCBD and SCBD vary between 0 and 1, beta regression was used to investigate the relationship between alpha diversity and LCBD and between SCBD and the number of sites occupied for each species (i.e., species occurrence) (Cribari‐Neto & Zeileis, 2010). The Kruskal–Wallis test was used to investigate differences in SCDB values between native (i.e. species occurring originally in Italian waters) and exotic (i.e. species originating from outside Italy) species.

All analyses were performed in R software version 3.4.3 (R Core Team, 2017). VIFs were checked using “car” R package (Fox & Weisberg, 2020), linear mixed models were fitted through the “lme4” R package (Bates et al., 2015), the model selection was performed with “AICcmodavg” R package (Mazerolle, 2019), the marginal and conditional *R* ^2^ were calculated with “partR2” R package (Stoffel et al., 2021), and the beta regression was performed with “betareg” R package (Zeileis et al., 2021).

## 3 RESULTS

**FIGURE 2:**
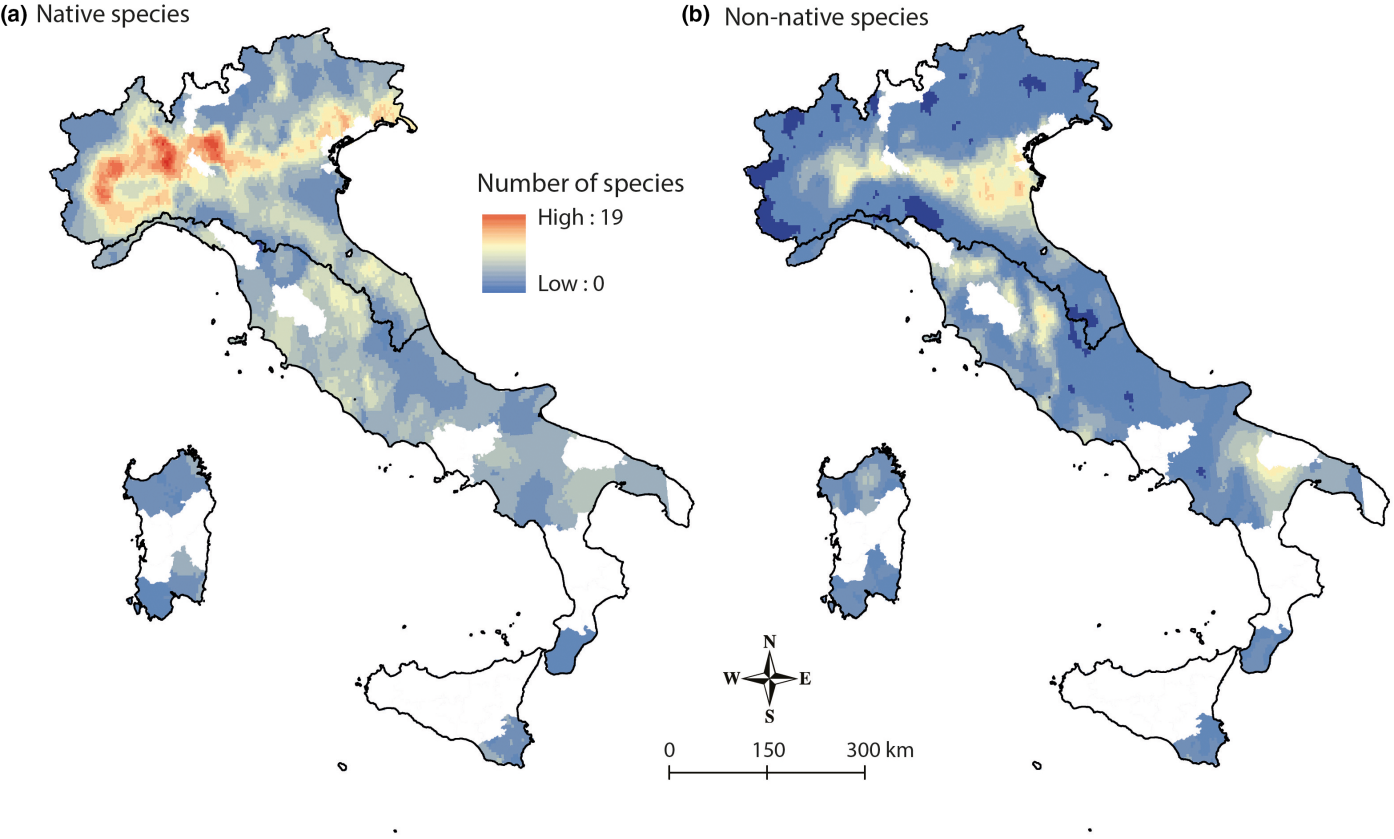
Native (a) and non‐native (b) alpha diversity (i.e., number of species) of fish communities in Italian inland waters. White areas represent zones for which no fish data was collected.

A total of 98 different fish species (of which 36 non‐native) were found in Italian rivers (Table S1). The highest values of alpha diversity were found in Northwest Italy, in the Padano‐Veneto district (PDV) where alpha diversity peaked at 27 species sampled in a single site, whereas the Island district (ISL) showed the lowest alpha diversity values (Figure 1). Native and non‐native species diversity showed different distributions, but both with hotspots in Northern Italy (Figure 2). BD_total_ was 0.7726 and LCBD values ranged from 0.00016 to 0.00042 among sites. According to beta regression, LCBD and alpha diversity were significantly related with a positive relationship (Pseudo‐*R* ^2^: 0.017, *p* < .001, Table 2a, Figure S1a).

**TABLE 2:** Results of beta regression analyses of (a) local contribution to beta diversity (LCBD) and (b) species contributions to beta diversity (SCBD) as response variables.

a) LCBD	Explanatory variables	Estimate	ES	*z*	*p*‐values	Pseudo‐*R* ^2^
	(Intercept)	2.116	0.00099	−2137.84	<.001	0.017
	Alpha diversity	0.00254	0.00032	8.02	<.001	
	Alpha diversity^2^	−0.00015	0.00002	−7.40	<.001	

b) SCBD	Explanatory variables	Estimate	ES	*df*	*p*‐values	Pseudo‐*R* ^2^
	(Intercept)	−1.826	0.0256	−71.32	<.001	0.727
	Species occupancy	0.00104	0.00009	11.51	<.001	
	Species occupancy^2^	−0.0000003	0.00000006	−4.574	<.001	

**FIGURE 3:**
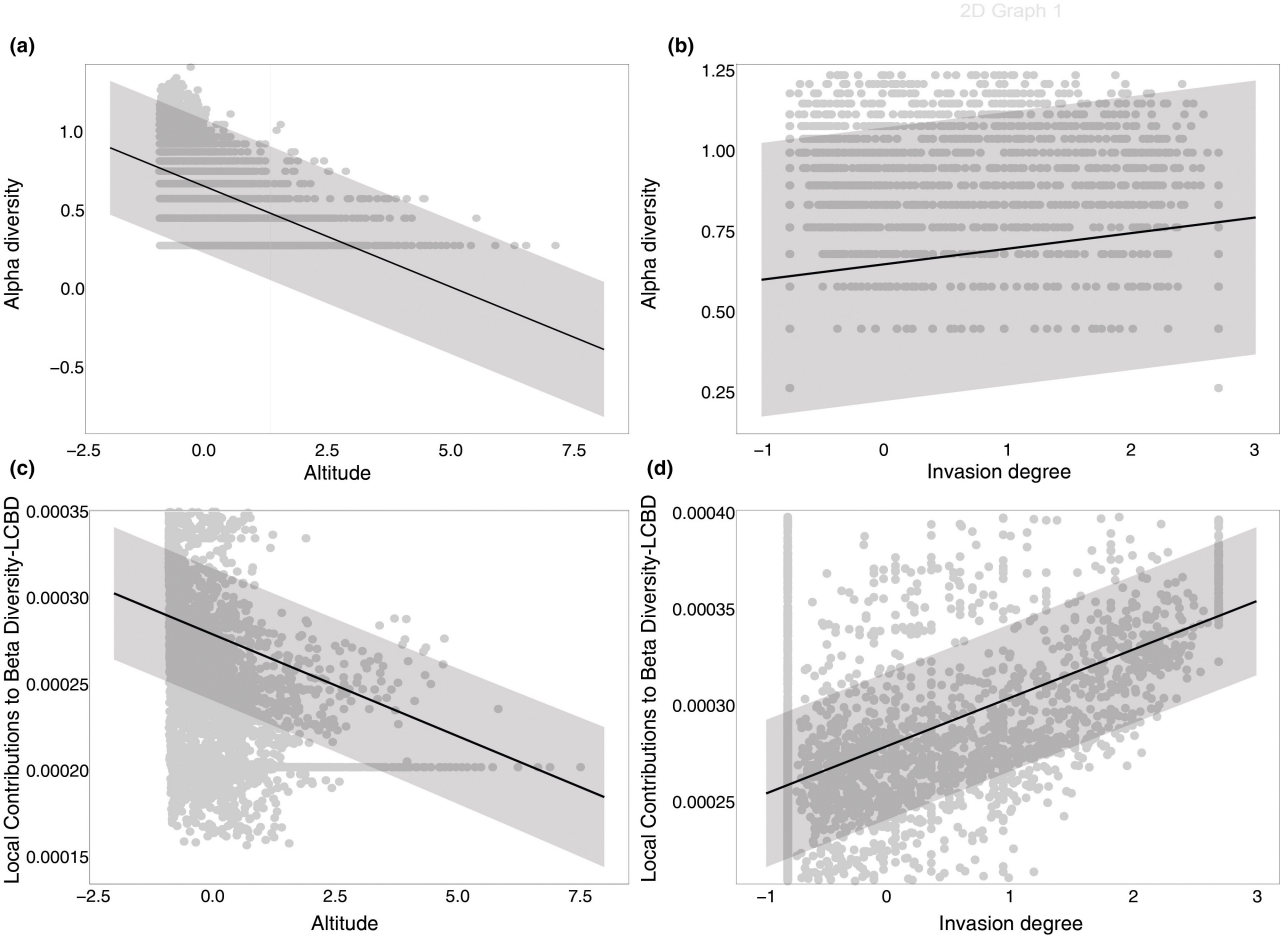
Main drivers of alpha diversity (a, b) and local contributions to Beta diversity – LCBD (c, d) predicted by linear mixed models (black lines) with 95% confidence interval (gray shading). Alpha diversity was log‐transformed, and the explanatory variables were standardized. Data points are also shown with gray dots (*n* = 3734). For model details see Table 3.

For alpha diversity, four land use features (artificial surface, rice field, forest, and freshwater), two geospatial variables (altitude and latitude), and the invasion degree were identified as best variables and included in the linear mixed model (conditional *R* ^2^: 0.482, marginal *R* ^2^: 0.389, Table 3a, Table S2a). Alpha diversity was significantly negatively affected by forest cover and altitude, whereas invasion degree, artificial surface, rice field, freshwater, and latitude related positively with alpha diversity (Table 3a, Figure 3a,b).

**TABLE 3:** Summary of linear mixed model results for (a) alpha diversity and (b) local contribution to beta diversity (LCBD).

	Explanatory variables	Estimate	ES	*df*	*t*	*p*‐values
a) Alpha diversity	(Intercept)	0.664600	0.052190	2	12.734	<.01
	Invasion degree	0.048410	0.003674	3774	13.174	<.001
	Latitude	0.038280	0.006567	1688	5.828	<.001
	Altitude	−0.128400	0.003866	3774	−33.213	<.001
	Freshwater	0.009861	0.003483	3774	2.831	<.01
	Forest	−0.036210	0.004001	3775	−9.049	<.001
	Rice field	0.032880	0.003509	3773	9.371	<.001
	Artificial surface	0.015340	0.003625	3773	4.231	<.001
b) LCBD	(Intercept)	0.000281	0.000019	2	14.449	<.001
	Invasion degree	0.000025	0.000001	3772	32.331	<.001
	Latitude	−0.000007	0.000001	3383	−4.982	<.001
	Altitude	−0.000012	0.000001	3773	−13.927	<.001
	Marine water	0.000003	0.000001	3773	3.835	<.001
	Forest	−0.000005	0.000001	3773	−5.195	<.001
	Heterogeneous agricultural area	−0.000003	0.000001	3772	−4.563	<.001
	Rice field	−0.000004	0.000001	3772	−4.774	<.001
	Artificial surface	−0.000003	0.000001	3772	−4.193	<.001

*Note*: Model estimates, standard error (ES), *t*‐test, and *p*‐values are reported for each retained variable.

For LCBD, five land use features (artificial surface, rice field, heterogeneous agricultural area, forest, and marine water), two geospatial variables (altitude and latitude), and the invasion degree were included in the linear mixed model as best variables (conditional *R* ^2^: 0.536, marginal *R* ^2^: 0.266, *p* < .01, Table 3b, Table S2b). Only invasion degree and marine water land use were positively related with LCBD, whereas artificial surface, rice field, heterogeneous agricultural area, forest, altitude and latitude showed a negative relationship with LCBD (Table 3b, Figure 3c,d).

**FIGURE 4:**
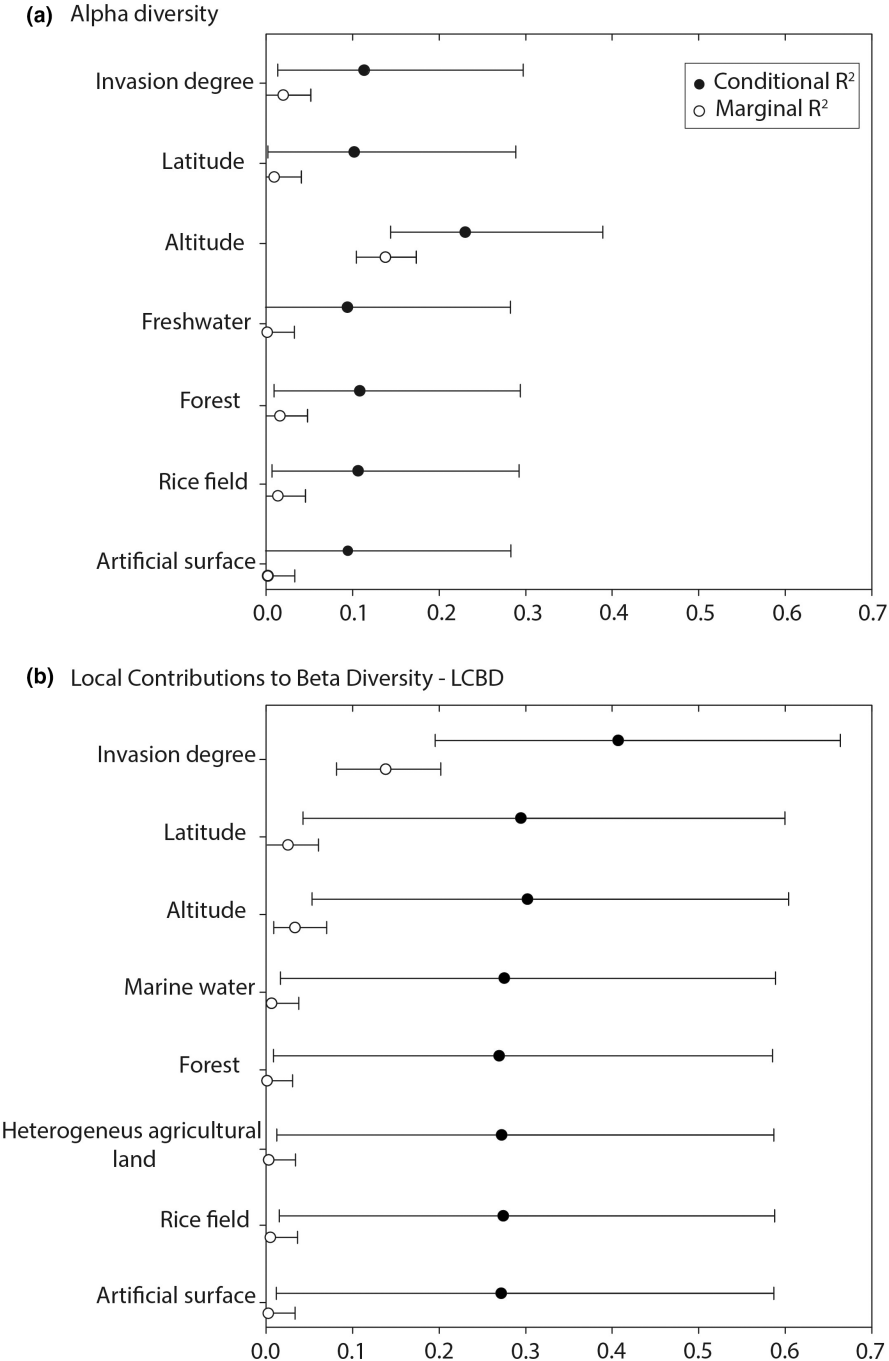
Conditional (black dots) and marginal (white dots) *R* ^2^ for predictors of alpha diversity (a) and local contribution to Beta diversity (b). Bars represent the confidence intervals at 95% estimated by 1000 bootstrap iterations.

In the alpha diversity model (Figure 4a), the partitioning of *R* ^2^ showed that altitude was the variable with the highest value of conditional and marginal *R* ^2^ (conditional *R* ^2^: 0.23 – IC: 0.14–0.39, marginal *R* ^2^: 0.14 – IC: 0.11–0.17) followed by invasion degree (conditional *R* ^2^: 0.11 – IC: 0.01–0.30, marginal *R* ^2^: 0.02 – IC: 0–0.05), forest, rice field, latitude, artificial surface, and freshwater variables (Table S3a).

In the LCBD model (Figure 4b), the invasion degree showed the highest conditional and marginal *R* ^2^ (conditional *R* ^2^: 0.41 – IC: 0.19–0.66, marginal *R* ^2^: 0.14 – IC: 0.08–0.203), followed by altitude (conditional *R* ^2^: 0.30 – IC: 0.05–0.60, marginal *R* ^2^: 0.03 – IC: 0.01–0.07), latitude, marine water, rice field, heterogenous agricultural land, artificial surface, and forest variables (Table S3b).

Total beta diversity was dominated by turnover (β_sim_) reaching 99.99% of total dissimilarity, whereas nestedness (β_nes_) accounted only for 0.01%. Both β_sim_ and β_nes_ were significantly related to invasion degree but with opposite trends: β_sim_ was positively related with invasion degree (*R* ^2^_adj_ 0.143, *p*‐value <.001) while β_nes_ was negative related with invasion degree (*R* ^2^_adj_ 0.06, *p*‐value <.001).

Species contributions to beta diversity was positively related to the number of sites a species occupied (Pseudo *R* ^2^: 0.727; *p* = <.001; Table 2b), with species with low occurrence contributing less to SCBD (SCBD ≤ 0.0001; Figure S1b). For example, the Adriatic sturgeon (*Accipenser naccarii*) which occurred at two sites had the SCBD value of 0.00004 (Table S1). Brown trout (*Salmo trutta* complex) and Italian chub (*Squalius squalius*) showed the highest SCBD values (0.19049 and 0.0688, respectively) and occurrence (1728 and 1703 sites out of 3734 sites, respectively). Italian native and exotic species did not differ in their SCBD values (KW *χ* ^2^ = 0.29, *df* = 1, *p* > .05).

## 4 DISCUSSION

This study examined the variation in alpha and beta diversity of fish communities in Italian rivers considering land use, geospatial variables, and invasion degree effects. As hypothesized (H1), invasion degree was the strongest driver for beta diversity and the second best driver of alpha diversity, after altitude. Alpha diversity, site uniqueness (i.e., LCBD), and the turnover component of beta diversity showed a positive relationship with invasion degree, whereas the nestedness component of beta diversity showed a negative relationship. In contrast to our hypothesis (H2), sites with unique fish communities (i.e. higher LCBD values) showed higher alpha diversity. The most widely occurring species contributed more to site uniqueness (i.e. SCBD), both for native and exotic species, disagreeing with (H3).

### 4.1 Invasion degree

We found a positive relationship between species richness and invasion degree suggesting that the presence of non‐native species equaled or exceeded species loss within sites (Li et al., 2020). However, the effect of environmental conditions cannot be ruled out, as environmental conditions can benefit both native and non‐native species increasing habitat complexity and, thus, provide habitats suitable for most native and non‐native species (Stohlgren et al., 2006), especially at low invasion degree (Takács et al., 2021). Furthermore, it is likely that a longer temporal scale, non‐native species could cause the loss of rare native fish species and diversity, resulting in change at a regional spatial scale (Dornelas et al., 2014; Moi et al., 2021).

Different results were found about non‐native species influence on alpha and beta diversity, depending, for example, on the study scale, river types, and diversity metrics used (Li et al., 2020; Takács et al., 2021). At the global level, non‐native species promote destabilization of native communities (Erős, Comte, et al., 2020) and contribute to fish extinction (Clavero & García‐Berthou, 2005). However, up to now, no fish extinctions due to non‐native species were documented at national level in Italian freshwaters (Bianco & Ketmaier, 2015; IUCN, 2021), even if they promote the decline of native species populations (e.g. Carosi et al., 2017; Castaldelli et al., 2013; Giannetto et al., 2012; Milardi et al., 2018).

We also found that the turnover was the main component of beta diversity in the fish communities, and it positively related to invasion degree. This suggested that the replacement of some species by others is the main phenomenon occurring at a regional scale, and non‐native species were involved in the process. On the other hand, the replacement of native species with non‐native species concurs with the increase of similarity of communities and thus homogenizes the biota (Kortz & Magurran, 2019; Rahel, 2002). Similar homogenizing effects of exotic species were previously found in Italy (Gavioli et al., 2019), but species introductions were found also to decrease the functional diversity of host communities (Milardi et al., 2020; Shuai et al., 2018). These results could be also assessed by considering only those areas which have been affected by invaders (e.g. Milardi et al., 2018) or by comparing communities before and after species introductions (Olden, 2006). Unfortunately, due to the historical species introductions that took place long time ago, we could only analyze the introduction gradient over a large number of sites.

Surprisingly, we found a positive relationship between invasion degree and LCBD values, which suggests that invaded communities showed unique species composition. However, sites with unique species composition include both species‐rich sites having peculiar combinations of native and non‐native species and degraded sites (Legendre & De Cáceres, 2013). Such complexity of LCBD suggests that its results should be evaluated using caution.

### 4.2 Geospatial and land use variables

Among geospatial and land use variables, altitude had the strongest influence on alpha and beta diversity. The decrease of fish alpha diversity along with increasing altitude was not surprising and could be linked to temperature, stream gradient (e.g., change of depth and width), habitat fragmentation, or availability of natural resources (e.g. Askeyev et al., 2017; Jaramillo‐Villa et al., 2010), although some exceptions have been reported with an increase in fish diversity at higher elevation in South America (Carvajal‐Quintero et al., 2015).

In our study, the fish community showed a decrease of LCBD along the altitudinal gradient, suggesting that communities at low altitudes contribute more to beta diversity (i.e., are probably more unique and diverse). Especially at intermediate altitudes, the rivers had greater number of species as compared to fish communities in the higher mountains, possibly by providing more habitat and fewer invaded sites at lower elevation (Gavioli et al., 2019; Takács et al., 2021). According to the literature, LCBD shows different altitudinal trends depending on the taxa and areas considered. For example, a negative relationship between LCBD and elevation was found in the Thysanoptera order (Dianzinga et al., 2020), whereas a unimodal relationship was found in microbial groups (Teittinen et al., 2016; Yeh et al., 2019).

Many species show a latitudinal gradient of diversity, with a decrease from the equator to the poles (Rosenzweig, 1995), but in our study, despite the large latitudinal gradient considered, alpha diversity showed an opposite latitudinal trend with the highest richness in the northern rivers, where hotspot of diversity were already revealed by Gavioli et al. (2019) and Milardi et al. (2020). Overall, native and non‐native species diversity showed different spatial distribution suggesting a different contribution to the total alpha diversity. This highlighted the importance of considering both native and non‐native species status in diversity studies, especially when biological invasions are occurring. However, animal translocation by humans, habitat availability, and thermal optima could also contribute to the latitudinal diversity gradient (Elvira & Almodóvar, 2001; Magurran et al., 2011; Pelayo‐Villamil et al., 2015; Wang et al., 2020).

Land use variables did not have a negative effect on alpha diversity, but rather human presence (i.e., rice field and artificial surface variables) showed a positive influence. We further found a negative effect of artificial surface and agricultural lands on LCBD suggesting that human impact resulted in less unique sites in terms of species composition. These results are not surprising given also the role of humans in promoting fish diversity changes through habitat alteration and species introductions and translocation (Anas & Mandrak, 2021; Leprieur et al., 2008; Rahel & Smith, 2018). Similar results were also found in fish communities of Brazil where human modified areas were found to have a peculiar assemblage of species, increased fish β diversity (Leão et al., 2020), and also peculiar macroinvertebrate assemblages (Hawkins et al., 2015).

### 4.3 Relationship between alpha and beta diversity and species contributions to beta diversity

The relationship between alpha and beta diversity can have different directions and can be affected by multiple factors depending on taxa and habitat type (e.g. Giovâni da Silva et al., 2018; Heino & Grönroos, 2017; Szabo et al., 2019). In our study, sites with unique species composition had high species richness. However, high alpha diversity and high LCBD values do not necessary mean higher ecological value of a site. For example, alpha diversity does not consider the native and non‐native status of species, and endemic and rare species could be underestimated by the presence of common introduced species such as common carp (*C. carpio*) or crucian carp (*Carassius spp*.,) (Gavioli et al., 2019).

Common and abundant species also drive SCBD values, which are directly linked to species occurrence (Cai et al., 2018; Giovâni da Silva et al., 2018; Heino & Grönroos, 2017). For example, the common species as brown trout (*Salmo trutta complex*) and Italian chub (*Squalius squalus*) showed high SCBD despite their widespread in the study area. Furthermore, SCBD values did not differ between native and exotic species, perhaps due to the simplified native and exotic fish communities, or due to the similar diversity contribution between natives and exotics. However, some exceptions to this result can be found in upland rivers where native species contribute more to diversity than exotic ones (Gavioli et al., 2019) because of different response of stream and river communities to the impacts (Erős, Czeglédi, et al., 2020).

## 5 CONCLUSIONS

Our study provides novel evidence that invasion degree plays a central role in shaping alpha and beta diversity patterns, and its effects could be stronger than other anthropogenic effects. Although the presence of non‐native species can increase local diversity, at the regional level, fish communities become more homogenous with the loss of endemic and rare species (Pool & Olden, 2012). In this scenario, it is crucial to prevent new species introductions and illegal release of fish (Rahel & Smith, 2018). In future studies, it will be important to evaluate separately the native and the non‐native components of communities to identify linkages between invasion dynamics and diversity loss of native assemblages.

## AUTHOR CONTRIBUTIONS

**Anna Gavioli:** Conceptualization (equal); data curation (equal); formal analysis (equal); investigation (equal); methodology (equal); validation (equal); visualization (equal); writing – original draft (equal); writing – review and editing (equal). **Marco Milardi:** Conceptualization (equal); data curation (equal); investigation (equal); methodology (equal); supervision (equal); writing – original draft (equal); writing – review and editing (equal). **Janne Soininen:** Investigation (equal); methodology (equal); writing – review and editing (equal); writing – original draft (equal); supervision (equal). **Elisa Soana:** Investigation (equal); data curation (equal). **Mattia Lanzoni:** Data curation (equal); investigation. **Giuseppe Castaldelli:** Resources (equal); supervision (equal); conceptualization (equal); data curation (equal), funding acquisition (lead); writing – original draft (equal); writing – review and editing.

## CONFLICT OF INTEREST

All authors claim no conflict of interest.

## Supporting information


Figure S1

Table S1

Table S2

Table S3

## Data Availability

Data included in this paper are already published and publicly available, original papers are referenced in the text.
