# Supplementary figures and images for: How does invasion degree shape alpha and beta diversity of freshwater fish at a regional scale?
Source: Ecol Evol. 2022 Nov 8;12(11):e9493. doi: 10.1002/ece3.9493 (PMC9643121; doi:10.1002/ece3.9493)

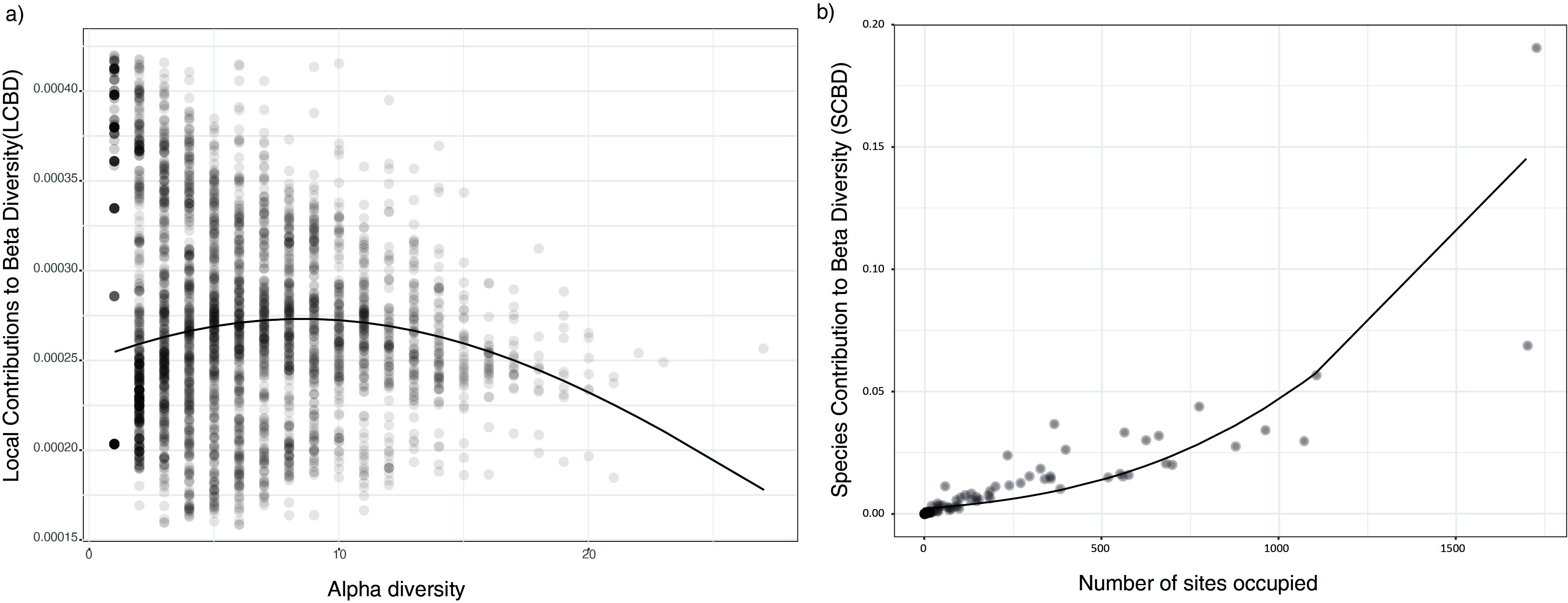

Supplement: Supplementary file 1 — Figure S1 [file ECE3-12-e9493-s002.jpg]
