# Supplementary material for: How does invasion degree shape alpha and beta diversity of freshwater fish at a regional scale?
Source: Ecol Evol. 2022 Nov 8;12(11):e9493. doi: 10.1002/ece3.9493 (PMC9643121; doi:10.1002/ece3.9493)
Supplement: Supplementary file 3 — Table S2 [file ECE3-12-e9493-s004.docx]

Supplementary Table 2: Summary of AIC results for LME models selection correlating alpha diversity (a) and LCBD (b) with altitude and latitude variables, invasion degree and land uses (abbreviations: NatNaked= Natural cover without vegetation, HetAgri_Areas= Heterogeneous agricultural areas, Winter crops= Non-irrigated arable land, Summer crops=Irrigated arable land). The “District” variable was included as a random effect. The number of estimated parameters for each model (K), the Akaike’s information criterion (AIC), the delta AIC (ΔAIC), the Akaike weights (AICcWt) and the Cumulative Akaike weights (CumWt) are shown.

| a) | Component model | K | AIC | ΔAIC | AICWt | CumWt |
| --- | --- | --- | --- | --- | --- | --- |
| A7 | Artificial surfaces+Rice fields+Forests+Freshwaters+Altitude+Latitude+Invasion degree | 10 | -957.36 | 0 | 1 | 1 |
| A6 | Artificial surfaces+Rice fields+Forests+Freshwaters+Marine waters+Altitude+Latitude+Invasion degree | 11 | -946.12 | 11.24 | 0 | 1 |
| A5 | Artificial surfaces+Rice fields+Forests+NatNaked+Freshwaters+Marine waters+Altitude+Latitude+Invasion degree | 12 | -936.68 | 20.68 | 0 | 1 |
| A4 | Artificial surfaces+Rice fields+Pastures+Forests+NatNaked+Freshwaters+Marine waters+Altitude+Latitude+Invasion degree | 13 | -925.51 | 31.85 | 0 | 1 |
| A3 | Artificial surfaces+Rice fields+Tree crops+Pastures+Forests+NatNaked+Freshwaters+Marine waters+Altitude+Latitude+Invasion degree | 14 | -914.15 | 43.21 | 0 | 1 |
| A2 | Artificial surfaces+Winter crops+Summer crop+Rice fields+Tree crops+Pastures+Forests+NatNaked+Freshwaters+Marine waters+Altitude+Latitude+Invasion degree | 16 | -907.54 | 49.82 | 0 | 1 |
| A0 | Artificial surfaces+Winter crops+Summer crop+Rice fields+Tree crops+Pastures+HetAgri_Areas+Forests+NatNaked+Freshwaters+Marine waters+Altitude+Latitude+Invasion degree | 17 | -902.11 | 55.25 | 0 | 1 |
| A1 | Artificial surfaces+Summer crop+Rice fields+Tree crops+Pastures+HetAgri_Areas +Forests+NatNaked+Freshwaters+Marine waters+Altitude+Latitude+Invasion degree | 16 | -894.38 | 62.98 | 0 | 1 |
| A8 | - | 3 | 1047.49 | 2004.85 | 0 | 1 |
| b) | **Component model** | **K** | **AIC** | **ΔAIC** | **AICWt** | **CumWt** |
| L1 | Artificial surfaces+Rice fields+HetAgri_Areas+Forests+Marine waters+Altitude+Latitude+Invasion degree | 11 | -64891.5 | 0 | 0.99 | 0.99 |
| L7 | Artificial surfaces+Rice fields+Forests+Freshwaters+Altitude+Latitude+Invasion degree | 10 | -64882.4 | 9.13 | 0.01 | 1 |
| L6 | Artificial surfaces+Rice fields+Forests+Freshwaters+Marine waters+Altitude+Latitude+Invasion degree | 11 | -64872.2 | 19.32 | 0 | 1 |
| L5 | Artificial surfaces+Rice fields+Forests+NatNaked+Freshwaters+Marine waters+Altitude+Latitude+Invasion degree | 12 | -64845.9 | 45.6 | 0 | 1 |
| L4 | Artificial surfaces+Rice fields+Pastures+Forests+NatNaked+Freshwaters+Marine waters+Altitude+Latitude+Invasion degree | 13 | -64817.6 | 73.93 | 0 | 1 |
| L3 | Artificial surfaces+Rice fields+Tree crops+Pastures+Forests+NatNaked+Freshwaters+Marine waters+Altitude+Latitude+Invasion degree | 14 | -64792.8 | 98.68 | 0 | 1 |
| L2 | Artificial surfaces+Winter crops+Summer crop+Rice fields+Tree crops+Pastures+Forests+NatNaked+Freshwaters+Marine waters+Altitude+Latitude+Invasion degree | 16 | -64767.8 | 123.77 | 0 | 1 |
| L0 | Artificial surfaces+Winter crops+Summer crop+Rice fields+Tree crops+Pastures+HetAgri_Areas+Forests+NatNaked+Freshwaters+Marine waters+Altitude+Latitude+Invasion degree | 17 | -64740.3 | 151.25 | 0 | 1 |
| L8 | - | 3 | -63542.5 | 1349.07 | 0 | 1 |
