# Supplementary material for: How does invasion degree shape alpha and beta diversity of freshwater fish at a regional scale?
Source: Ecol Evol. 2022 Nov 8;12(11):e9493. doi: 10.1002/ece3.9493 (PMC9643121; doi:10.1002/ece3.9493)
Supplement: Supplementary file 4 — Table S3 [file ECE3-12-e9493-s003.docx]

Supplementary Table 3: Conditional and marginal R2 for predictors of alpha diversity a) and Local Contribution to Beta Diversity b). Confidence intervals (CI) at 95% were also showed.

| a) | **Conditional R2** | | **Marginal R2** | |
| --- | --- | --- | --- | --- |
|  | *R2* | *CI* | *R2* | *CI* |
| Altitude | 0.23 | 0.14-0.39 | 0.14 | 0.11-0.17 |
| Invasion degree | 0.11 | 0.01-0.30 | 0.02 | 0-0.05 |
| Forest | 0.11 | 0.01-0.29 | 0.02 | 0-0.48 |
| Rice field | 0.11 | 0.01-0.29 | 0.01 | 0-0.05 |
| Latitude | 0.1 | 0-0.029 | 0.01 | 0-0.04 |
| Artificial surface | 0.09 | 0-0.28 | 0 | 0-0.03 |
| Freshwater | 0.09 | 0-0.028 | 0 | 0-0.03 |
| b) | **Conditional R2** | | **Marginal R2** | |
|  | *R2* | *CI* | *R2* | *CI* |
| Invasion degree | 0.41 | 0.19-0.66 | 0.14 | 0.08-0.203 |
| Altitude | 0.3 | 0.05-0.60 | 0.03 | 0.01-0.07 |
| Latitude | 0.29 | 0.04-0.6 | 0.02 | 0-0.06 |
| Marine water | 0.28 | 0.02-0.59 | 0.01 | 0-0.04 |
| Rice field | 0.27 | 0.01-0.59 | 0 | 0-0.04 |
| Heterogenous agricultural land | 0.27 | 0.01-0.59 | 0 | 0-0.03 |
| Artificial surface | 0.27 | 0.01-0.59 | 0 | 0-0.03 |
| Forest | 0.27 | 0.01-0.58 | 0 | 0-0.03 |
